# Experiences of Older Adults Receiving the Faith Community Nursing Model of Care in Australia

**DOI:** 10.1177/08980101251374374

**Published:** 2025-09-09

**Authors:** Catherine Paterson, Benjamin Kopp, Charissa Wright, Elise Button, Reegan Knowles, Mary-Ann Carmichael, Lemma N Bulto, Oluwaseyifunmi Andi Agbejule, Juliana Christina

**Affiliations:** Caring Future Institute, 375072 Flinders University and Central Adelaide Local Health Network, Bedford Park, Australia; 2234 University of Canberra, Canberra, Australia; Caring Future Institute, 568639 Flinders University, Australia; Caring Future Institute, 64767 Flinders University South Australia, Australia; Caring Future Institute, Flinders University South Australia, Australia; Universitas Jenderal Achmad Yani, Faculty of Health sciences and technology, Cimahi, Indonesia

**Keywords:** older adults, Group/Population, holistic nursing, faith community nursing, qualitative study, experiences, Australia

## Abstract

**Background:** The Faith Community Nursing (FCN) model of care is nurse-led spiritual or faith-integrated holistic care that has been provided in faith communities worldwide. Studies exploring individuals’ experiences within such models of care are limited. **Purpose:** To understand the experiences of older adults with chronic conditions who received care within an FCN model of care in Australia, led by registered nurses and supported by volunteers. **Design:** An exploratory qualitative approach. **Methods:** Face-to-face semistructured interviews were conducted with ten older adults with chronic conditions, recruited using purposive sampling. Interviews were digitally recorded and transcribed verbatim for the thematic data analysis process. **Findings:** Four main themes emerged: coping and faith integration, accepting care to meet daily living needs, social connections and respite care, and professional support and services. Older adults with chronic conditions in this study described how the FCN model of care met their needs and positively impacted their physical, emotional, and spiritual well-being. **Conclusion:** Positive experiences within the FCN model of care highlight the potential value and impact of such models of care on individuals and families in the faith community. Further studies exploring the effectiveness, broader application, and sustainability of the FCN model of care are warranted (198/200).

## Introduction

Every human being deserves holistic nursing care that encompasses all aspects of well-being, including spiritual health (Wells et al., 2024). The holistic nursing approach focuses on treating the whole person, addressing physical, emotional, social, and spiritual needs (Akyüz et al., 2024). Spiritual care is a holistic nursing principle that can be delivered by faith community nurses (FCN) (Hawthorne & Gordon, 2020). Faith community nursing, also known as parish nurses, provide nurse-led models of care for members of faith communities (Bergquist & King, 1994). Specifically, FCN endeavors to address spiritual or faith-based care needs across the lifespan to promote wellness, disease prevention, health promotion, and healing within a faith-based community (Paterson et al., 2021). Globally, the FCN model of care has been growing and gaining momentum since its inception in 1984, when Granger E. Westberg, a Lutheran pastor in the US, founded it (Paterson et al., 2021). “*Faith community nurses may still be referred to as a parish nurse, congregational nurse, health ministry nurse, crescent nurse, or health and wellness nurse to fit a community's culture and faith tradition*” (National Association of School Nurses & Amerian Nurses Association, 2005).

The role of FCN was introduced in Australia in 1997 (van Loon, 1998). Models of FCN are now offered in the Bahamas, Canada, Costa Rica, Finland, Germany, Ghana, India, Japan, Kenya, Korea, Madagascar, Malawi, Malaysia, New Zealand, Nigeria, Pakistan, Palestine, Philippines, Singapore, South Africa, Swaziland, Ukraine, United Kingdom, the US, Zambia, and Zimbabwe (Kruse-Diehr et al., 2021). A recent systematic review identified that irrespective of the location or the particular faith denomination, FCN reported they were able to address the physical, psychological, spiritual, congregational, communication, health system, and family-related needs of those in their care (Paterson et al., 2021). Faith community nursing also identified they were partnering with individuals, families, and healthcare teams, as well as increasing awareness and building connections in a spiritual manner (Paterson et al., 2021). Caring for diverse and holistic facets of the human experience is at the core of nursing.

While spirituality is unique to everyone, many do not identify with spirituality. However, connection, hope, meaning, and peace are often relevant to everyone (Miller et al., 2023). Religion and spirituality are considered important cultural factors that shape experiences, value systems, and behaviors, especially in the context of health and well-being (Bennett et al., 2024). Spirituality has been defined as “the connection that people make to something beyond themselves as a means of reaching self-actualization” (Savel & Munro, 2014, p. 276). Spirituality is different than religion, which can be defined as “a community of persons who share a particular set of beliefs about the transcendent along with shared practices, texts, rituals, and teaching” (Savel & Munro, 2014, p. 277). Spirituality is of particular importance to many people with critical chronic conditions, with the provision of spiritual care to guiding medical decision-making and providing comfort and community (Balboni et al., 2022). Spiritual belief and regular engagement in a faith community have been associated with better healthcare outcomes such as lower risk of mortality; less smoking, alcohol, and drug use; and improved quality of life (QoL) (Balboni et al., 2022). Despite this, spiritual needs are infrequently addressed in medical care (Balboni et al., 2022). Faith community nursing plays a central role in leading holistic models of care that address the unmet spiritual needs of people with serious illnesses or chronic conditions living in their community.

A systematic review critically synthesized the impact of FCN models of care on various health outcomes. However, this review (Kruse-Diehr et al., 2021) concluded that, given the limitations and weaknesses of the existing studies in the field to evidence FCN's impact on patient outcomes, this area of research remains in its infancy for individuals with various health conditions. Further research should be prioritized to provide robust qualitative and quantitative data in a range of patient groups in community settings to understand the experiences of receiving care from FCN models and interventions (Kalánková et al., 2021; Paterson et al., 2021). Faith community nursing is underpinned by a conceptual model (Figure 1) (Ziebarth, 2014) which clearly distinguishes the concept of the patient/nurse embedded within community settings with the attributes of faith integrating, health-promoting, disease-managing, coordinating, and empowering approach to accessing healthcare (Kruse-Diehr et al., 2021; Paterson et al., 2021). Faith appears to be an integrated and continuously occurring attribute in this model of nursing care (Ziebarth, 2014). Despite the growing international interest (Kruse-Diehr et al., 2021; Paterson et al., 2021) in FCN models of care, there remains a significant knowledge gap in peoples’ experiences of utilizing such community services across various health conditions.

**Figure 1.**
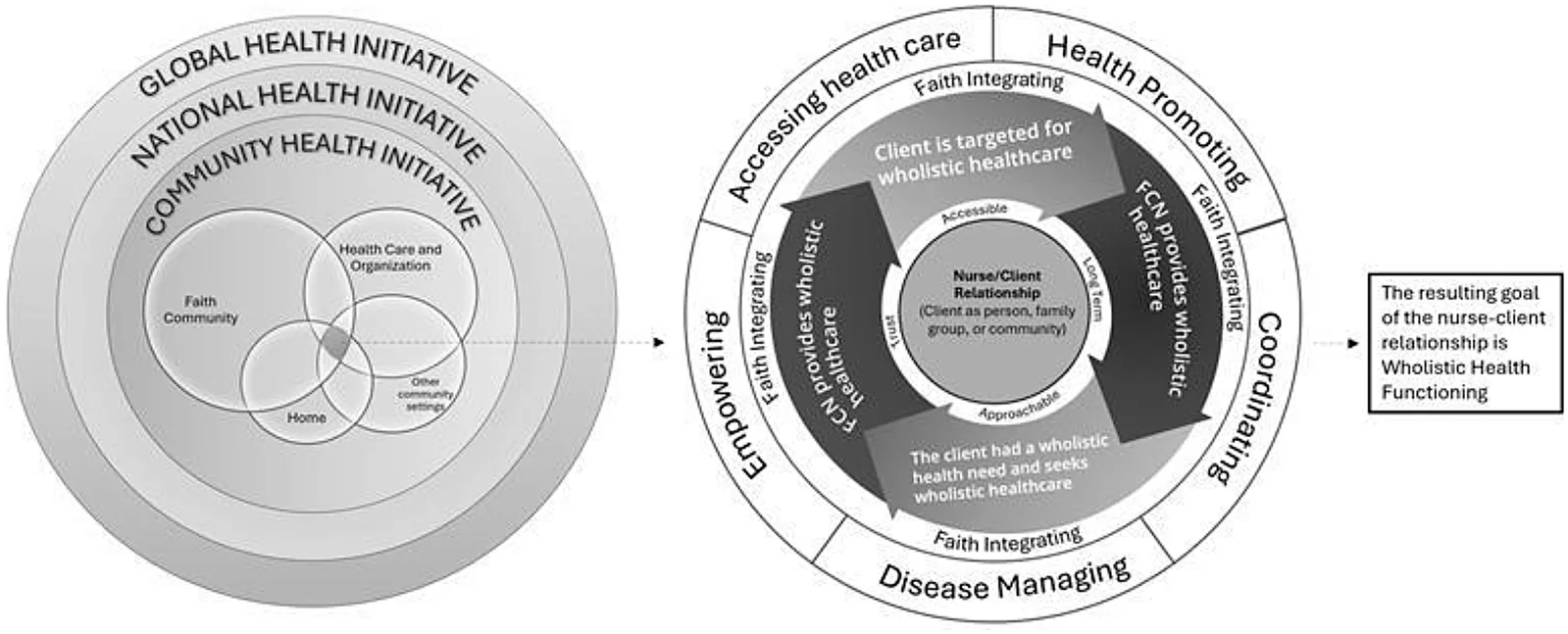
Faith community nursing (FCN) Conceptual Model.

## Aims

This qualitative study aimed to understand the experience of individuals who had received care within an FCN model of care in an Australian community. To achieve this objective, the study addressed the following research question: What are the experiences and perspectives of clients regarding their care within an FCN model of care?

## Methods

### Study Design

A qualitative study was undertaken to provide an exploration of the phenomenon of interest (Sandelowski, 2000). This approach garners direct, rich, and firsthand descriptions of a phenomenon, especially when there is limited published information available (Turale, 2020). This approach was selected to gain a comprehensive understanding of the perspectives of older adults who received care within the FCN model in a community setting. The reporting of this study adhered to the consolidated criteria for reporting qualitative studies checklist (Supplementary File 1).

### Ethical Considerations

This study conformed to the requirements of the Helsinki Declaration and was approved by the Human Research Ethics Committee of the University of Canberra (12106). All participants provided voluntary written informed consent and were assured of anonymity.

### Setting

The study was conducted between May 2023 and June 2024 in one FCN community organization in the Australian Capital Territory (ACT), Australia.

### Faith Community Nursing Model

The FCN Program is administered through a Holistic Care Nursing program as a community health initiative at a church in the ACT. This program is free of charge and managed by two registered nurses who are members of the Australian Faith Community Nurses Association and a team of volunteers. The FCN program aims to promote whole personal health for individuals and nurture positive relationships with the individual and other people, the individual and the created environment, and the individual and God. All activities seek to integrate faith and health based on the belief that health and well-being are primarily facilitated through the spiritual dimension of the individual. This program does not seek to replace other agencies. The care provided for individuals ensures a holistic approach to care. The Biblical foundation underpinning the program is “*he (Jesus) sent them to proclaim the kingdom and to heal*” (Luke 9:2, English Standard Version Bible, 2001).

#### Program Overview

The program commenced operation in October 2011 and has been operating weekly every Monday from 10 a.m. until 3 p.m. for over 10 years. This program offers a range of nursing care, including personal care, medication administration, health promotion, health screening checks, navigation of the wider healthcare system, and various volunteer-supported activities. A service that enables carers to have a break for rest and respite is also available. The program also provides free mobile hairdressers to come regularly.

The FCN model of care is inclusive of older adults from all faith denominations, for those living with any type of chronic condition(s) and provides respite for informal carers. Older adults were not required to belong to the faith community of the place of worship where this program was delivered, nor were they expected to join.

#### Nurses

Faith community nursing provides professional advice, education, and support for older adults and carers, liaisons with other services, and advocates for them when required. Nurses provided overall leadership of the program, including oversight of the volunteers. The nurses developed care plans with older adults and provided holistic nursing care including physical, psychological, social, and spiritual care. Nurses also provided symptom management, care coordination, and decision support, directed supportive care through home visits, telephone follow-up, and used advocacy and system navigation skills to provide mobile support for older adults and their informal carers. Nurses also helped older adults with funeral plans and discussed advance directives with families. When required they also liaised with community clergy.

#### Volunteers

There are approximately 30 volunteers associated with the church program. Some volunteers set up the worship space for the day and pack away all items after the program. Holistic Care Nursing Ministry owns a van with wheelchair/disability access which is available to transport older adults to and from the church if their carer cannot bring them. Older adults were also provided with mobility aids from stock freely available with HCNM. Volunteers used their vehicles to transport older adults. Two activities coordinators develop an annual plan of activities, including guest speakers, entertainers, quizzes, arts and crafts, pet therapy, games that are appropriate to the ability and mobility levels, and birthday celebrations. Kitchen volunteers provide morning tea, water and other refreshments. They also collect a two-course hot lunch donation and serve with volunteer assistance.

#### Spiritual Support

Included in each Monday's activities is a prayer time which usually takes place in the chapel led by the nurses and clergy. All older adults who wish to attend are encouraged to bring forward prayer items. The spiritual activities are nondenominational, of short duration, and participation is voluntary. There are opportunities for one-on-one spiritual support provided by a volunteer pastoral team.

### Participants

A purposive sampling method was used to recruit older adults who had accessed the FCN model of care for their chronic conditions. Eligible participants had to have received care from the FCN model of care irrespective of the length of time they had been receiving care, gender, or type of chronic condition. Participants were required to be 18 years of age or older and capable of engaging in written and verbal English communication.

### Data Collection

All data were collected through face-to-face semistructured interviews. An interview guide, informed by a systematic literature review on FCN (Paterson et al., 2021), and the conceptual model of FCN (Ziebarth, 2014) aimed to explore the research interests of the study (Table 1). Interviews were conducted by an experienced professor (with over 25 years of experience in health services research, including qualitative studies), and two research students. Prior permission was secured from the managers at the FCN church organization to conduct the study. The researchers had no prior relationships with any participants or members of the FCN church organization. Researchers’ names and contact information were displayed on advertisements posted in the community daycare area. Interested individuals contacted the researchers. In those meetings, the inclusion criteria received a participant information sheet and written informed consent form. Participants returned signed written informed consent to researchers in person when interviews were conducted. All participants were assured of anonymity, with personal information remaining confidential and unidentified. Before the interviews, each participant was verbally asked if they were still agreeable to participating in this study.

**Table 1.** Interview Topic Guide Questions

Can you tell me about your care you have received at Covenant Care? **Perception of care** Want to know: why they think this, any examples as evidence of what they state, how did receiving this care makes them feel? **Perceptions of health professionals involved care** Can you tell me what you think about the care you receive from the FCN involved in your care? Want to know: if patients think these FCN have adequate knowledge about their condition (explore groups individually), why they think that, examples of instances where professional knowledge has helped / not helped the patient, how helpful are the professionals / why? What information have you been provided with about you care/condition? Want to know: what the patient thinks about the information, did they use any of it—if so why, if not why not, what information have they received from the different professionals involved in their care? **Experience and reporting of additional concerns/supportive care needs out with Covenant care** Have you called/visited any of your healthcare providers regarding any other issues? (GP/ Hospital/ Out of hours/ community Nurses/other). If so why? What was discussed at this time? What happened as a result of your call/visit? Were you happy with the outcome of your call/visit—if yes or no, why? How did the outcome make you feel? **Holistic Care** **Probe: Psychological, Physical, Spiritual, and Social experiences of support** In general, how do you feel about the support you receive from your healthcare providers/ Covenant Care over the last few weeks? How do you feel about the level of contact you have had with health professionals/covenant over the last few weeks? Is there any other aspect regarding the support you have received in the last few weeks that you would like to discuss? **General Closing:** Thank you for sharing your story and experiences with me. It's been really interesting to hear about. Is there anything else about your experiences that you would like to talk to me about? Is there anything you feel we have missed or you would like to tell me? Thank you very much for your time today, I really do appreciate it …

Face-to-face interviews were conducted onsite in private rooms to create a safe space. During the interviews, all participants were encouraged to share their personal experiences of receiving FCN care and provide relevant additional comments. Ten participants were interviewed, and interview durations ranged from 28 to 45 min. The interviews were digitally audio-recorded and resulted in approximately 470 pages of transcription.

### Data Analysis

The interviews were transcribed verbatim and analyzed by authors using the thematic analysis method. One researcher listened to the audio recordings to gain an initial understanding of the context. The analysis was conducted manually (without any software package) and guided by Braun and Clarke's thematic analysis steps (Konstantinos, 2024). The first author then thoroughly read and reread all interview transcripts, and a line-by-line inductive coding process was conducted. Statements that represented similar words and meanings were grouped into categories to identify potential themes and subthemes. All authors independently reviewed the themes, subthemes, and representative quotes for accuracy and provided feedback. Regular meetings between authors were held to review and refine the potential themes and subthemes. The themes were carefully defined and refined to capture the essence of each interview. This thematic analysis process generated four main themes, each of which was provided with examples of relevant statements.

### Rigor

Trustworthiness and rigor were considered (Milne & Oberle, 2005). Credibility was ensured by peer debriefing in the research team during the analysis process. Dependability was ensured by having a debriefing session among all the interviewers to maintain consistency in data collection. All the interview transcripts were quality-checked for accuracy. Confirmability was considered by keeping an audit trail throughout the process, including documenting postinterview reflective notes, and during the analysis to make an accurate record of all the decisions and processes undertaken during the study.

## Findings

### Participants Characteristics

Of the 11 potential participants attending the FCN Program, 10 consented to take part in the study. The 10 participants were included in the final analysis. No reason was provided by the one older adult who did not want to participate in the study. There were four males (40%) and six females (60%), with a mean age of 80.9 years (ranging from 72 to 90 years), and most were married (70%). Chronic conditions experienced by participants were Alzheimer's disease (*n* = 2;11%), atrial fibrillation (*n* = 1;5%), cataracts (*n* = 1;5%), dementia (*n* = 2;11%), diabetes (*n* = 1; 5%), heart disease (*n* = 4;21%), pacemaker use (*n* = 1;5%), hip and knee replacements (*n* = 1;5%), lymphedema (*n* = 1;5%), macular degeneration (*n* = 1;5%), mobility problems (*n* = 1;5%), neurogenerative disease (*n* = 1;5%), shoulder replacement (*n* = 1;5%), and stroke (*n* = 1;5%) (Table 2).

**Table 2.** Participant Characteristics

Gender	Marital status	Age	Long-term health conditions
Female	Married	77 years	Shoulder replacement, atrial fibrillation, stroke
Male	Married	77 years	Alzheimer's disease
Female	Married	76 years	Neurogenerative disease, macular degeneration
Female	Single	72 years	Alzheimer's disease
Female	Married	79 years	Dementia
Male	Married	83 years	Heart disease, mobility problems, lymphedema, cataracts
Male	Married	82 years	Diabetes, heart disease, dementia
Male	Married	90 years	Heart disease
Female	Widow	82 years	Stroke, mobility problems
Female	Widow	88 years	Hip and knee replacements, heart disease, pacemaker

The thematic analysis identified four themes: (1) coping and faith integration, (2) accepting care to meet activities of daily living needs (ADLs), (3) social connection and respite care, and (4) professional supports and services, which were all intertwined with living with chronic conditions. Quotations representing participants’ experiences and views on the FCN program were provided to support the themes.

### Theme 1: Coping and Faith Integration

The first theme demonstrated coping and faith integration support provided by FCN for older adults with chronic conditions in this study. Participants described experiencing severe emotional distress, anxiety, and depression following life-changing events due to deteriorating health. Three participants reported that developing resilient coping strategies was incredibly challenging, particularly in facing advanced age and multiple health issues. Strong reliance on faith and spiritual guidance to navigate through difficult times was viewed as important. Support during difficult times was needed, and FCN was crucial in providing nonjudgmental support and encouraging and supporting the integration of faith, as highlighted by this participant:

“I had the first stroke and that was absolutely demoralizing and it was hell. But yes, I didn’t want to be here anymore, and I tried suicide and that didn’t work. … they said, we don’t judge you, we just love you, we know things have got very bad. And I said, look, I’ve had the worst anxiety, fear that you can’t imagine. I said I felt like I’d lost my life. Well, I had, because I was a grandma carer, and I wasn’t allowed to do that anymore, according to my daughter. She said, you can’t do it. So she said, I love you, Mum, we’ll come and see you. She did, she came to see me every day” (P1, Female, 77 years).

Faith in God was a helpful coping mechanism and strength for many. Participants expressed that perceiving that they were not alone in this world and that there was a higher purpose was comforting. Participants demonstrated resilience by finding innovative ways to cope with and adapt to their changed circumstances. FCN provided an environment that modeled trust which was comfortable and provided acceptance that was felt by all participants. The environment improved their psychological well-being and helped them to develop positive resilience strategies. Two participants claimed:

“I may be repeating myself, but you go to school, then you go to college, you go here and there, and there's pressure on you. And here, there's no pressure, I just feel comfortable. If I don’t, no one as yet has told me, smacked my hand or done anything. I just feel comfortable, which is what it's all about” (P2, Male, 77 years).

“You come here, there are other people within the same age path. You see them trying and it does make you want to try a bit harder. Well, that's me. A lot of them are not giving up. They're still going and still trying” (P9, Female, 82 years).

Participants were empowered by a newfound ability to ask clarifying questions and engage in conversation with spiritual meaning. They embraced and believed in the life–death–life worldview, which was about connecting with others, finding strength on their journey to cope and ultimately surrendering to God, as stated by one participant:

“I walk with God and my life with God is such that I have to let myself be led by him. And I said, God, I’d be happy if you’d take me off, there are times where I’d rather go home, meaning Heaven, that's my ultimate thing. But it's not my time” (P10, Female, 88 years).

All participants reported that attending the FCN program provided a sense of belonging and spiritual support, which participants highly valued. Not all the participants shared the same religious backgrounds and had different interdenominational experiences. However, they reported that accepting different faith practices within the community fostered inclusivity, understanding, and mutual respect in shared activities and prayers. Three participants sought spiritual healing and miracles for their chronic conditions. Participants valued rituals such as lighting candles for prayer needs, which provided a tangible way to express their faith. Such rituals were found to help participants feel connected to their community and their spirituality.

“I just love them, they have a time of prayer needs, that's what the candles are about, and they light candles for different ones that have prayer needs. And she can sit and pray for them, because we spoke about … [the FCN] says a simple prayer, a covering prayer. But it's not the way that we would do it, but that's all right, I just accept their faith” (P1, Female, 77 years).

“I said to God yesterday in church, I said a little prayer, they were talking about receiving a healing. I said, God, I can believe for it, I can believe for it every time, we’ve spoken about … But they talk about healing a lot, they believe in miracles, and they happen for some people, they get their miracles” (P4, Female, 76 years).

“I sometimes come in and light candles in here during a session. But I haven’t been along to church for a long time. I come here with this group” (P8, Female, 82 years).

There was a complex interplay between personal desires, and community support, and emphasizing the role of faith and spirituality in the lives of participants. This underscored the importance of inclusivity and adaptability, in maintaining connections for a harmonious spiritual experience.

### Theme 2: Accepting Care to Meet ADL Needs

The second theme described the experiences of older adults in accepting care and services to meet their ADL needs. Participants experienced the chronic condition's impact on the basic ADLs needs such as personal hygiene, eating and drinking, dressing, toileting, and mobility. They also required assistance to fulfil the nonessential activities for day-to-day functioning or instrumental ADL (IADLs), including house cleaning and maintenance. Two of them explained the transition and impact of “adjusting” to accepting help from others, including those within the program. Help was important in addressing both ADLs and IADLs needs and varied from accepting help from house cleaners, receiving assistance with self-care (eating, drinking, toileting, washing, and dressing), and attending HCNM program, which initially was challenging for some, but acknowledged to be essential for well-being. The profound impact of cognitive and physical degenerative diseases on the participants’ lives highlighted their resilience and the unique challenges they faced. Two participants stated:

“Our daughters got on his case and said, Dad, you’re so stingy with your money, why won’t you pay a cleaner? He said, oh, I hate to have to do that. They said, Dad, you’ve got the money, you have got such a good pension, you and Mum put together, you can afford a cleaner. So we started at HCNM and they got on our case again and then they said, now you need a cleaner” (P1, Female, 77 years).

“I did the usual things I used to do, like washing the cars and doing the gardening, duh-duh, duh-duh-duh. When I was doing the gardening, if I can say, I just got a sensation, not physical, but mental, and I just had to give it up. I just gave it up. It was stressful” (P2, Male, 77 years).

Due to advancing age and physical and mental impacts secondary to chronic conditions, ongoing and daily adjustments were required to make adaptations to new realities, often associated with loss, bereavement, and frustration. Two participants expressed that they adjusted slowly over time as they needed to acknowledge their boundaries and maintain safety. Participants were self-managing their chronic conditions and frequently needed to balance their activities carefully to avoid overexertion, especially four with heart conditions. The importance of having supportive family and community members was central to facilitating this transition due to the emotional toll of losing cherished ADLs and IADLs. Participants also reported that if they required any practical aids to assist with ADL needs, such as walking sticks, wheelchairs, hoists, or commodes, these were available from the HCNM program.

“Not being able to do all the things I used to do, because I can’t. I used to play the piano, write, read, all those activities, none of them I can do anymore, very frustrating” (P3, Female, 76 years).

“The archdeacon here is a very good person with everybody. And if you need anything … a walking stick or a chair or something you might need … coming here, it makes me get up. It makes me get here, it makes me dress properly, I have people to talk to, and it does me good. I go home and I feel good, after I’ve been” (P10, Female, 88 years).

### Theme 3: Social Connections and Respite Care

Social connections and respite care were found to have a great emotional impact on participants. Participants expressed a profound loss due to the death of friends and loved ones. Due to friendships that lasted for many years, individuals reported that it was difficult to return to places that reminded them of deceased friends. The profound emotional impact of losing long-term friends and reducing social connections was highlighted by one participant who lost a friend nearly 90 years old. She stated:

“But I’ve lost my girlfriends that … Well, one, she died about five months ago, she was 94. And I’d known her since I was five” (P10, Female, 88 years).

Companionship and camaraderie were highly valued within the FCN program, with participants expressing joy in spending time with existing and new friends within this community as a sense of “belonging” and “integration.” Friendships were further developed by participating in activities and developing shared interests, like games, which helped to build and strengthen these connections. The difference in social dynamics, such as having more male or female friends did not matter, as friendships were based on individual circumstances and social connections. Two participants highlighted their individuality and the challenge of integrating into larger social settings due to their solitary identities which required effort to engage in communal activities. However, the community environment helped alleviate feelings of isolation, particularly for those who lived alone or had limited mobility. Finding solace, emotional support, and understanding among peers and within the FCN community was important to the 10 participants. The presence of familiar faces and the establishment of routines contributed to the participants’ sense of belonging.

“And there's a couple of guys here I’ve really connected with, and there's one guy … and he has been such a blessing to all of us. He comes and shares about the train trips he's taken” (P1, Female, 77 years).

“And here, when you see them every week, they become your close friends. And everybody's interested how you feel, and what they can do for you. And that's important, because when you’re getting older and you’re not feeling well, you think, well, why, what will I do? Or something. And as I said, every Monday, I know I come here, I get up, I get dressed properly, and I get here” (P10, Female, 88 years).

Additionally, the FCN program provides informal respite care for carers and family members to support their social needs. Participants expressed this service enabled their loved ones to have a “break” to catch up with their friends, do housework, allow relaxation time, pursue hobbies, personal care, or other activities. Two participants with dementia expressed the main reason they attended the FCN program was to provide respite care for their husband whom they treasured. Respite care helped to prevent feelings of fatigue, frustration, and the need for regular rest to prevent burnout. Many others articulated that it provided them with a bit of “space” and different “company” also from each other which was often needed.

“[The FCN program] it gives me a break and gives him a break. He [husband] is so much happier now he's got today to himself. He also meets with the Veterans once a week for coffee, they’re mates, and he hangs out just for the coffee morning” (P1, Female, 77 years).

“My husband, who is also my carer, gets a day off. I don’t know what he's doing now, but he gets a few hours where he doesn’t have to worry about me at all, because everything I need is here. So, that is good for him, because as you can imagine, it's very tiring for him, and frustrating. And my experience here is always a very pleasant one, it's very pleasant. And they’re very attentive and it's just quiet and nice” (P3, Female, 76 years).

Carer rest was perceived to be important among most of the participants. One participant noted that the FCN service also facilitated further respite care services which were an important support in navigating access to wider healthcare services.

### Theme 4: Professional Support and Services

This final theme of professional support and services described participants’ experiences regarding the support and services they received from the FCN program. All the 10 participants reported diverse experiences and perspectives of their involvement with the FCN program. There was a clear emphasis on the friendliness, kindness, or nonjudgmental nature of the community in all the participants. Participants valued the quality care and professionalism provided to them, and they praised the nurses and volunteers for their competence and dedication in supporting them. Participants described their interactions with the FCN as being well-trained, disciplined, and devoted to providing excellent care underpinned by empathy and faith. They raised the need for regular visits to specialists and doctors as part of managing their chronic conditions. Three participants felt they did not get satisfactory answers or solutions but the nurse was able to provide clarification, navigation, and disease management support.

“The holistic side of it's nice. We get a prayer service. That's quite nice, the holistic side of it” (P6, Male, 83 years).

“They’re all loving, caring, gentle people, and that's very nice. Because out there in the community, when you’re in a wheelchair life is very difficult, but here they understand” (P3, Female, 76 years).

“The company, and they entertain us, and they’re great people, and they look after us. They’ve even got a nurse here, they can get me into the bathroom” (P5, Female, 79 years).

Participants stated that the FCN was vital to their lives, indicating long-term engagement and deep integration into weekly routines. There was a strong emphasis on the relationships built in partnership with staff who were seen as caring, understanding, and receptive to their individual needs. Participants voiced that they appreciated being accepted as they were, without pressure to conform or change, irrespective of their religious denomination. There were also opportunities to engage in different activities such as Tai chi, bingo, and social games, which were appreciated and found to be engaging for participants. These activities were viewed as opportunities for learning new skills or hobbies, even if participants did not master them. All were encouraged to try new things and engage at their own pace but only if they wanted to. This helped to create a sense of independence and self-esteem by managing certain aspects of their lives with the support of the FCN.

Some participants expressed that they desired more opportunities for physical activity, such as walking around the church and gardens. The participants themselves acknowledged that due to the limited mobility among the group that attended the FCN program, and the need for staff supervision, it was challenging to organize such activities.

## Discussion

This qualitative study provides valuable insight into the experiences of older adults with chronic conditions within an FCN model of care in Australia. Data from interviews reflected that participants’ engagement within the FCN model supported coping, adjustment to changes in daily living needs, provided respite to carers, enabled social connection, and strengthened faith in the context of long-term health conditions. Participants also reported that the FCN program led to therapeutic relationships with providers (nurses and volunteers). This was demonstrated by increased trust, a perceived sense of increased approachability, accessibility of support and care, and long-term engagement. Participants praised the program's comfortable, accepting environment; and the caring, understanding, accessible, and receptive nature of the staff. Acceptance of participants, irrespective of their religious denomination, further fostered trust and approachability. Overall, all participants expressed a high degree of satisfaction with their experiences and resulting well-being, with reported program benefits extending beyond the patient to informal carer respite for family members. These findings demonstrate the value of the FCN model of care for study participants.

Findings from this qualitative study reporting participants’ experiences within an FCN model of care, align with concepts presented in the FCN conceptual model (Ziebarth, 2014). Participants relayed how the FCN program was rooted in faith and spirituality; provided support with disease management; empowered them by offering practical aids, emotional support, training on self-advocacy, and access to healthcare; and provided coping strategies and education on essential concepts, encouraging them to adapt their routines for better health outcomes. Faith community nursing was part of a coordinated approach to addressing holistic well-being for older adults and their carers. These supports align with the FCN conceptual model that emphasizes that a faith-based model of care must include the five components of coordinating, empowering, accessing healthcare, health-promoting, and disease managing and must be underpinned by faith integration.

The integration of faith through prayer, ritual, and spirituality in the FCN model of care, was valued by participants who universally expressed positive experiences regarding connecting or reconnecting with their faith or spirituality, and how this connection led to a stronger tie to the community. Spiritual well-being is recognized as a determinant of health contributing to QoL (Balboni et al., 2022). The findings in this study indicated that a sense of community, spirituality/faith, and belongingness in the model may have provided participants with the confidence to use faith/spirituality as a coping strategy. Connecting to a person's spiritual beliefs has been shown to increase resilience and improve well-being (Bennett et al., 2024; Miller et al., 2023) which could lead to older adults feeling hopeful and more empowered. While the experiences of the nurses (or volunteers) were not explored in this research, studies have shown there is benefit to clinicians as well as patients when addressing spiritual dimensions of care (Merath et al., 2020). Research has shown that connecting to patients and their spiritual needs can help to build trust and lead to stronger connections (Balboni et al., 2022; Miller et al., 2023). However, spiritual care requires nurses (or those who provide care) to be aware of their own beliefs, the patients’ beliefs and how they intersect (Miller et al., 2023; Savel & Munro, 2014).

The evolution of the biomedical model in healthcare towards a patient-centered biopsychosocial model (Byrne et al., 2020), has led to recognition of the importance of holistic aspects of a person, including spirituality. Spiritual well-being of patients is integral to patient-centered care (Savel & Munro, 2014) and recommendations encourage nurses and other healthcare professionals to incorporate it into their clinical practice (Merath et al., 2020). Despite acknowledging that patient-centered care should be responsive to individual needs, patients still experience that their spiritual and emotional needs are often neglected (Quinn & Connolly, 2023). With increasing demands and scarcity of resources in modern healthcare settings, the FCN model of care in this study is one approach that could help to support the needs of older adults with illnesses in faith communities. The professional discipline of nursing has evolved from a sacred spiritual self-caring (Watson, 2022). The nursing role encompasses assessing, responding, and advocating for the cultural and spiritual needs of patients, families, and communities, using a holistic approach to care (Barnum, 2006). There are calls for greater awareness of the benefits and increased provision of spiritual care from all healthcare professionals including nurses (The Lancet RegionalHealth Europe, 2023). There is potential scope for the FCN model of care to involve medical and allied health professionals to meet holistic nursing care, particularly for the spiritual needs of older adults within their faith-based communities.

## Implications for holistic nursing practice and FCN

As understandings of whole person health and well-being continue to evolve. There have been calls to reform the way healthcare is traditionally delivered, particularly holistic nursing care, to incorporate practice models that involve community outreach to address the entire continuum of patient needs (Dyess et al., 2016). The findings of this qualitative study indicate that the FCN program was a beneficial model of care that leveraged volunteers in the faith-based community to provide holistic, patient-centered care. The findings indicate that such a community-based, nurse-led, faith-based model of care can effectively address the spiritual and emotional needs of patients, aspects that are often overlooked in holistic nursing care delivery. Study findings also align with existing literature, with evidence demonstrating that positive individual and population holistic care health benefits arise from intentionally collaborating with faith-based entities (Kruse-Diehr et al., 2021).

Older adults within the FCN model of care who participated in this study experienced chronic conditions with a high potential of progressing to become more critical illnesses. Critical chronic conditions can trigger an existential crisis, with patients questioning the meaning and purpose of life (Balboni et al., 2022). The importance of spirituality is well-documented for older people with critical conditions and those nearing the end of life (Balboni et al., 2022). Provision of spiritual care should be extended to include all patients who desire it, not merely those facing serious or terminal illness (Armitage, 2023). Unanswered questions remain regarding the application of the FCN model of care for younger people or in other areas of healthcare (i.e., women and children, mental health, addiction, cancer).

## Implications for holistic nursing science and FCN

The study results are promising in support of the FCN model of care, which optimized the well-being of older people with critical chronic illness, through spiritual and holistic support. The FCN model aligns with the calling of the church to meet people's spiritual and physical needs, and the purpose of the nursing profession to care for the whole person.

The findings of this small qualitative study are encouraging and warrant further research on the integration of holistic nursing and FCN models of care. Focus should be placed on the applicability to other faiths, populations, contexts, and settings. The impact of involvement in the FCN models of care from volunteers’ perspectives should also be explored. Costing studies are needed to understand the resources required to replicate a similar FCN program in various settings, inform scale and spread, and future research. A key question remains around the sustainability of a model of care run solely through volunteers, at a time when participation in formal volunteering in Australia is declining (Australian Institute of Health and Welfare, 2023; Volunteering Australia, 2024). Future research of integrated holistic nursing and FCN models of care should include hybrid implementation-effectiveness studies that demonstrate the impact on clinical outcomes (The Lancet Regional, 2023) and which components of the model are implementation/sustainable and are most valued by older adults in various contexts (Garner, 2022). Additionally, it is important to define appropriate outcome measures aligned with the integration of holistic nursing and the FCN conceptual model and appeal to funders (to reduce reliance on volunteers). Demonstrating the effectiveness and sustainability of FCN models of care will facilitate the broader implementation of such models in various communities.

## Strengths and Limitations

The strengths of this study include the rich, detailed descriptions of participants’ experiences of FCN across different health conditions, which provide rigorous data and valuable insights into the needs and perspectives of an often-underrepresented age group in research. These insights are valuable for understanding faith-based care and its impact on well-being, as well as informing the development of similar programs in other faith communities and settings. Limitations include a single site, a small sample size, and Caucasian ethnicity. Additionally, it was impossible to distinguish between comments relating to the overall program, nurse and or volunteer roles, and meaning that results cannot be attributed to particular aspects of the FCN Program.

## Conclusions

Positive experiences within the FCN model of care highlight the potential value and impact of such models of care on individuals and families in faith communities. Integration of faith through prayer, ritual, and spirituality, an important component of the FCN conceptual model, was valued by older adults with chronic conditions. Further studies exploring the effectiveness and implementation, broader application to various populations, and sustainability of the FCN model of care are warranted. Consideration should be placed on the inclusion of medical and allied health professionals within similar models of care.

## Supplemental Material

sj-pdf-1-jhn-10.1177_08980101251374374 - Supplemental material for Experiences of Older Adults Receiving the Faith Community Nursing Model of Care in Australia Supplemental material, sj-pdf-1-jhn-10.1177_08980101251374374 for Experiences of Older Adults Receiving the Faith Community Nursing Model of Care in Australia by Catherine Paterson, Benjamin Kopp, Charissa Wright, Elise Button, Reegan Knowles, Mary-Ann Carmichael, Lemma N Bulto, Oluwaseyifunmi Andi Agbejule and Juliana Christina in Journal of Holistic Nursing
